# Synergistic effects of combined breathing training and aerobic exercise on cardiopulmonary function in chronic heart failure: a systematic review and meta-analysis

**DOI:** 10.7717/peerj.20954

**Published:** 2026-03-18

**Authors:** Mingli Li, Mingcong Xie, Wei Qi, Feiyun Song, Feng Guo, Mingyun Sun

**Affiliations:** College of Physical Education, Anqing Normal University, Anqing, Anhui, China

**Keywords:** Breathing exercises, Aerobic exercise, Chronic heart failure, Cardiopulmonary function

## Abstract

**Background:**

Chronic heart failure is a complex clinical syndrome that poses a serious threat to human health. Breathing training and aerobic exercise are key strategies for promoting rehabilitation in these patients. The present study aimed to investigate the effects of breathing training combined with aerobic exercise on cardiopulmonary function and quality of life in patients with chronic heart failure.

**Methods:**

A computer-based search was conducted using a combination of subject terms and free-text terms to identify randomized controlled trials evaluating the effects of breathing training combined with aerobic exercise on cardiopulmonary function in patients with chronic heart failure from both Chinese and international databases. All databases were searched from inception to April 2025. Two reviewers independently screened studies, extracted data, and assessed the risk of bias. Meta-analyses were performed using RevMan 5.4 and Stata 17.0.

**Results:**

A total of seven randomized controlled trials involving 246 patients were included. The analysis showed that, compared with the control group, breathing training combined with aerobic exercise resulted in statistically significant improvements across several functional outcomes, including exercise duration (SMD = 0.32, 95% CI [0.05 to 0.60], *p* = 0.02) and quality of life (SMD = −1.09, 95% CI [−1.78 to −0.40], *p* < 0.001). No significant effects were observed on cardiac function parameters. Subgroup analyses suggested that factors such as sex composition may influence intervention effects, and sensitivity analyses confirmed the robustness of these findings.

**Conclusion:**

Breathing training combined with aerobic exercise has positive effects on pulmonary function, cardiopulmonary exercise capacity, and quality of life in patients with chronic heart failure, while its impact on cardiac function parameters appears to be limited. Given the limited number and quality of the included studies, these conclusions and the optimal intervention duration require further confirmation in high-quality research. This study has completed registration with the Systematic Review Program at PROSPERO under registration number CRD420251014242.

## Introduction

Chronic heart failure (CHF) is a clinical syndrome caused by structural or functional heart diseases that impair ventricular filling and/or ejection capacity. It is a systemic disease characterized by high morbidity and mortality, poor quality of life, and high treatment costs, and it poses serious threats to life at multiple levels (Adamopoulos et al., 2014). Studies have shown that the prevalence of chronic heart failure is continuously increasing. The mortality rate has remained close to 40–50% over the past five years, and the incidence after the age of 65 is approximately 10 per 1,000 (Roger et al., 2011). As a result, chronic heart failure has become a serious global public health problem. Improving outcomes in patients with heart failure has become a major focus in the healthcare field (Baodi et al., 2018). Therefore, improving prognosis, slowing disease progression, and enhancing quality of life are key goals in the rehabilitation of patients with chronic heart failure.

The main symptoms of chronic heart failure include dyspnea, orthopnea, paroxysmal nocturnal dyspnea, and peripheral edema. These symptoms are usually caused by reduced cardiac output and excessive fluid retention (Brake & Jones, 2017). In addition, Jiang et al. (2001) reported that patients with chronic heart failure may also experience mental health–related disorders, such as depression. These symptoms not only reduce exercise capacity and limit daily activities but also impair the quality of life of patients. Therefore, improving cardiopulmonary function and quality of life has become an important component of the comprehensive management of patients with chronic heart failure.

In clinical practice, strategies to improve exercise tolerance in patients with chronic heart failure include cardiac resynchronization therapy, optimized pharmacological treatment, various exercise training programs, cardiac rehabilitation, and individualized management strategies. These interventions can improve cardiopulmonary function and prognosis to some extent (Chan & Tsang, 2019; Bjarnason-Wehrens et al., 2020). However, in older patients with chronic heart failure, the overall effects of these interventions remain limited because of reduced cardiopulmonary reserve and the presence of multiple comorbidities.

In recent years, in addition to standard clinical care, physical exercise has been widely shown to be an effective approach to improving physical fitness and reducing morbidity (Chircop & Jelinek, 2006). Studies have demonstrated that regular aerobic exercise can reduce mortality and rehospitalization rates in patients with chronic heart failure and improve peak oxygen uptake (VO_2_peak), skeletal muscle abnormalities and quality of life (Corrà et al., 2002; Conraads et al., 2004; Zhou et al., 2023). At the same time, several studies and meta-analyses have shown that combining resistance training with exercise interventions has advantages over single exercise modalities in improving exercise capacity and maximal oxygen uptake (Jesus et al., 2020; Alshamari et al., 2023). However, because of impaired respiratory muscle function and reduced ventilatory efficiency, exercise training alone has limited effects on improving respiratory function.

Furthermore, patients with chronic heart failure commonly experience abnormal respiratory function, dyspnea, and exercise intolerance (Haykowsky et al., 2007; Jing & Ning, 2016). Respiratory muscle training can improve respiratory muscle strength and endurance, reduce breathing frequency, and increase blood oxygen saturation. These effects help improve cardiopulmonary function and enhance exercise tolerance in patients (Laoutaris et al., 2004). Previous studies suggest that breathing training combined with aerobic exercise may produce synergistic effects in improving cardiopulmonary function and exercise capacity in patients with chronic heart failure. However, most existing studies are small randomized controlled trials with limited sample sizes, and their results remain inconsistent. In addition, there is a lack of systematic synthesis and evaluation of the available evidence.

## Data and Methods

### Research methodology

This systematic review followed the Preferred Reporting Items for Systematic Reviews and Meta-Analyses (PRISMA 2020) guidelines. This systematic review was prospectively registered with PROSPERO (registration number: CRD420251014242) prior to study selection. The protocol is available at: (https://www.crd.york.ac.uk/PROSPERO/recorddashboard).

### Inclusion and exclusion criteria for literature

#### Inclusion criteria

(1) Research type: Randomized Controlled Trial (RCT); (2) Research subjects: Patients clinically diagnosed with chronic heart failure, aged ≥18 years; (3) Intervention measures: The intervention measure for the experimental group was breathing training combined with aerobic exercise (IMT+AT), while that for the control group was aerobic exercise (AT/AIT/AET). (4) The search time limit is from the establishment of each database to April 2025. (5) The experimental result indicators selected include sustained PImax (SPImax), maximal inspiratory pressure (PImax), and minute ventilation carbon dioxide output slope (VE/VCO2 slope), left ventricular ejection fraction (LVEF), left ventricular end-diastolic diameter (LVEDD), left ventricular end-systolic diameter (LVESD), 6-minute walking distance (6MWD), exercise time, Quality of life (Qof).

#### Exclusion criteria

(1) Repeatedly published literature; (2) Conference abstracts and literature for which the full text cannot be obtained; (3) Literature not in English or Chinese; (4) Systematic review or meta-analysis; (5) Animal experiments.

### Retrieval strategy

We systematically searched the following electronic databases from their inception to search end date: PubMed, Ovid, Embase, Web of Science, Cochrane Library, CNKI, Wanfang, VIP, and Sinomed. The search strategy combined subject terms and free-text terms. Search terms related to the effects of breathing training combined with aerobic exercise on patients with chronic heart failure included: Heart Failure OR Cardiac Failure OR Heart Decompensation OR Congestive Heart Failure OR Right Sided Heart Failure OR Left Sided Heart Failure OR Myocardial Failure (b) Breathing Exercises OR Respiratory Muscle Training OR Muscle Training, Respiratory OR Training, Respiratory Muscle (C) Exercise* OR Physical Exercise* OR Aerobic Exercise* OR Isometric Exercise* OR Acute Exercise* OR Exercise Training* OR Physical Activit*. (The detailed search strategy is provided in Appendix 1).

### Literature screening and data extraction

EndNote X9 software (Clarivate, Philadelphia, PA, USA) was used to import and manage the retrieved search results. Two researchers (L-ML and X-MC) independently screened the titles and abstracts according to predefined inclusion and exclusion criteria. Full texts of potentially eligible studies were then reviewed for final selection and cross-checked. Any disagreements were resolved by a third reviewer (S-MY). For the studies finally included, data were independently extracted by the two researchers using a standardized, predesigned Excel data extraction form. The extracted information included authors, year of publication, study design, basic participant characteristics, intervention type and duration, outcome measures, tools used for quality assessment, whether sample size calculation was reported (yes/no), breathing training devices and their specifications, and whether measurement-related information (such as reliability or reproducibility indices, if available) was reported (Tables 1 and 2).

**Table 1:** General characteristics of the included studies.

First author	Year	Age (years)	Sample size	Sex (male/ female)	Inclusion criteria	Exclusion criteria	Sample size calculation reported (Y/N)
Adamopoulos	2014	T:57.8 ± 11.70	21	T:19/2	Patients were aged >18, stable moderate to severe CHF with systolic dysfunction (LVEF ≤35.00%), NYHA functional class II–III, and a signed informed consent (compatible with each centre’s ethics committee regulations)	Pulmonary limitation (FVC <60.00% predicted or FEV_1_/FVC <60.00%), active smoking, recent thoracic surgery, CCS class III–IV angina, corticosteroid treatment, substance abuse, cognitive impairment, inability to perform exercise testing, and major non-cardiac conditions affecting survival.	N
		C:58.3 ± 13.20	22	C:17/5			
Winkelmann	2009	T:54.0 ± 12.00	12	T:7/5	Patients with stable chronic heart failure attributable to left ventricular systolic dysfunction (left ventricular ejection fraction <45.00%) and inspiratory muscle weakness (PImax <70.00% of predicted). All patients had a diagnosis of chronic heart failure for more than 6 months and had no hospital admission or changes in medication during the previous 3 months.	History of pulmonary disease, current smoking, angina, recent myocardial infarction or cardiac surgery (<6 months), orthopedic or neurological disease, treatment with steroids, or cancer chemotherapy.	N
		C:59.00 ± 9.00	12	C:4/8			
Laoutaris	2021	T:68.10 ± 4.30	20	T:20/0	Patients were aged 18–80 years with stable,symptomatic chronic heart failure classified as NYHA class II–III, receiving optimal medical treatment, and with a left ventricular ejection fraction ≤35.00%.	Uncontrolled arrhythmia, pulmonary edema orpulmonary congestion within the previous 30days, cognitive, neurological, or orthopedic limitations, respiratory infection within 30 days before study initiation, and pulmonary limitations (e.g., chronic obstructive pulmonary disease)	N
		C:64.80 ± 6.44	18	C:16/2			
Trevizan	2021	T:56.00 ± 3.00	9	T:7/2	Patients aged 30–70 years with NYHA class II–III heart failure, LVEF ≤40.00%, VO_2_peak ≤20.00 mL⋅ kg^−^^1^⋅ min^−^^1^, BMI ≤35.00 kg/m^2^, and optimized medical therapy were included.	Comprised recent myocardial infarction or cardiac surgery (<6 months), unstable angina, pacemaker, CRT or ICD implantation, atrial fibrillation, smoking, pregnancy, severe pulmonary disease, neurologic or orthopedic disease,malignancy, dialysis-dependent chronic kidneydisease, insulin-dependent diabetes mellitus, regular exercise participation, and medication changes or hospitalization during the study.	N
		C:57.00 ± 2.00	12	C:7/5			
Sadek	2024	T:51.80 ± 8.30	10	T:5/5	Male and female patients aged 45–65 yearswith chronic heart failure diagnosed for morethan 6 months, left ventricular ejection fraction (EF) ≤45.00%, and New York Heart Association (NYHA) functional class II–III were eligible, provided there had been no hospital admission or changes in medication during the previous 3 months.	Pulmonary limitation (forced expiratory volume and/or vital capacity <60.00% of predicted),o rthopedic or neurological disease, history of significant cardiac arrhythmia, myocardial infarction or cardiac surgery within the past 6months, unstable or poorly controlled blood pressure, and end-stage heart failure.	N
		C:51.6 ± 13.80	10	C:5/5			
Sadek	2022	T:51.80 ± 8.30	10	T:5/5	Male and female patients aged 45–75 yearswith NYHA class II–III chronic heart failure, LVEF ≤45.00%, and inspiratory muscle weakness (MIP <70.00% predicted), diagnosed for >6 months without recent hospitalization or medication changes, were included.	Comprised pulmonary limitation (<60.00% predicted), orthopedic or neurological disease, significant arrhythmia, recent myocardial infarction or cardiac surgery (<6 months), unstable blood pressure, and end-stage heart failure.	N
		C:51.6 ± 13.80	10	C:5/5			
Yuan	2022	T:62.50 ± 4.22	50	T:24/26	Met the diagnostic criteria for chronic heart failure according to the Chinese Guidelines for the Diagnosis and Treatment of Heart Failure (2018).with stable symptoms and signs for more than 3 months.New York Heart Association (NYHA) functional class II–III;Aged 55–69 years.	Severe cognitive impairment or those unable to cooperate;Limb dysfunction;Severe arrhythmia;Unstable angina pectoris, acute myocardial infarction;Uncontrolled hypertension.	N
		C:61.32 ± 4.50	50	C:23/27			

**Notes.**

marginal notes T Experimental group C Control group Y reported N Not reported

Note. Adamopoulos et al., 2014; Winkelmann et al., 2009; Laoutaris et al., 2021; Trevizan et al., 2021; Sadek et al., 2024; Sadek et al., 2022; Yuan et al., 2022.

**Table 2:** Characteristics of the interventions included in the study.

First author	Mode of movement	Intervention time and frequency	Respiratory training device	Device specifications	Intervention cycle	Outcome index	Jadad score	Measurement reliability (ICC/SEM/MDC)
Adamopoulos (2014)	60% SPImax IMT+AT	3 times a week, 30 min IMT+45 min AT	TRAINAIR^®^ (Project Electronics Ltd, UK)	Pressure load range: 0 to −300cmH_2_O Pressure accuracy: ±0.1%,Electronic pressure manometer Computer-based biofeedback software 2-mm air leak toprevent glottis closure.	12 weeks	①②③④⑤ ⑥⑦⑨	3	c
	10% SPImax IMT+AT							
Winkelmann (2009)	30% PImax IMT+AT	7 times a week 30 min IMT+3 times a week AT	Healthscan (Products Inc., Cedar Grove, NJ, USA)	Fixed pressure threshold system	12 weeks	③⑧⑨	3	c
	AT	3 times a week Target 20 min, increasing by 5 min every 2 weeks until exercise reaches 45 min						
Laoutaris (2021)	60% SPImax IMT+AT	3 times a week, 30 min IMT+30 min AT	TRAINAIR^®^ (Project Electronics Ltd, UK)	Flow-resistive loading system	12 weeks	①②③④⑤ ⑥⑦⑧⑨	3	c
	AT	3 times a week, 30 min AT+30 aerobics						
Trevizan (2021)	60% MIP IMT+AT	5 times a week, 30 min IMT +3 times a week, 60 min AT	POWERbreathe Plus^®^	Resistance set as % of maximal inspiratory pressure (MIP)	16 weeks	②⑤⑨	3	c
	AT	3 times a week, 60 min AT						
Sadek (2024)	60% MIP AET+IMT	3 times a week, 30 min AET+IMT	PowerBreathe^®^	High-intensity IMT at 60% MIP	12 weeks	⑤⑥⑦⑧	5	c
	AET	3 times a week, 30 min AET						
Sadek (2022)	AET+IMT	3 times a week, 30 min AET+IMT	PowerBreathe^®^	Resistance-based inspiratory muscle training	12 weeks	④	5	c
	AET	3 times a week, 30 min AET						
Yuan (2022)	AET+IMT	3 times a week, 45 min AET+20-30 min IMT	N	N	12 weeks	②④⑧⑨	2	d
	AT	3 times a week, 45minAT						

**Notes.**

marginal notes ① SPImax ② VE/VCO_2_slope ③ PLmax ④ exercise time ⑤ LVEF ⑥ LVESD ⑦ LVEDD ⑧ 6MWD ⑨ quality of life AT/AIT/AET Aerobic Exercise IMT Breathing exercises c reliability not reported but cited d no reliability information

All outcome measures were assessed using standardized and validated methods as reported in the original studies. Detailed measurement specifications are provided in the “Appendix 4” section.

Note. Adamopoulos et al., 2014; Winkelmann et al., 2009; Laoutaris et al., 2021; Trevizan et al., 2021; Sadek et al., 2024; Sadek et al., 2022; Yuan et al., 2022.

### Study selection and data extraction reliability

To ensure consistency and methodological rigor, two researchers (L-ML and X-MC) conducted study selection and data extraction according to standardized procedures. Differences at each stage were resolved through discussion, and a third reviewer (S-MY) was consulted when necessary. Independent data extraction was performed for a subset of the included studies to verify consistency, and all discrepancies were resolved by consensus.

### Literature quality evaluation

**Figure 1:**
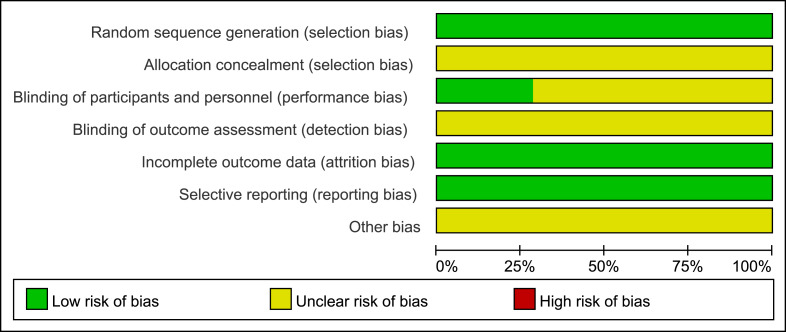
Risk of bias.

**Figure 2:**
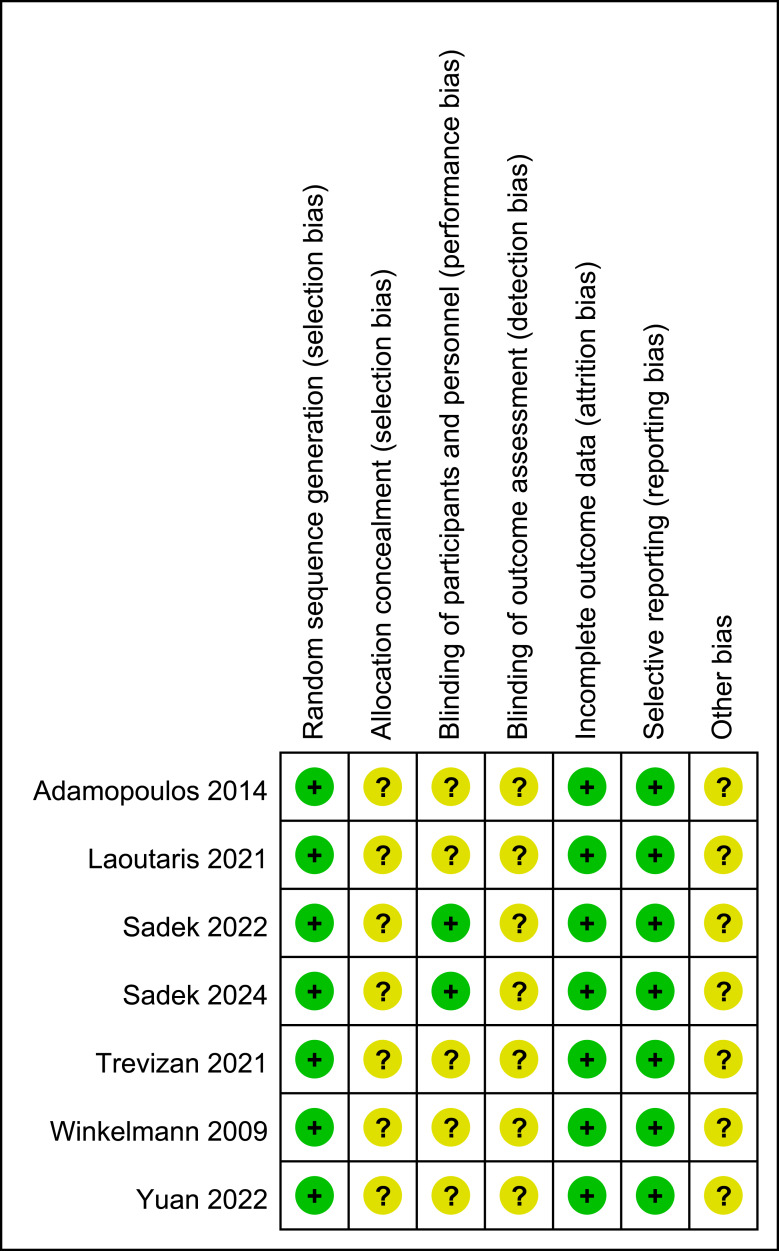
Risk of bias summary. Note. Adamopoulos et al., 2014; Laoutaris et al., 2021; Sadek et al., 2022; Sadek et al., 2024; Trevizan et al., 2021; Yuan et al., 2022; Winkelmann et al., 2009.

Two researchers (L-ML and X-MC) independently assessed the methodological quality of the included studies using the Cochrane Handbook–recommended risk of bias tool for randomized controlled trials (Figs. 1 and 2).

The evaluation domains included random sequence generation, allocation concealment, blinding of participants and personnel, blinding of outcome assessors, completeness of outcome data, risk of selective outcome reporting, and other potential sources of bias. If a study fully met the above criteria, it was considered to have a low risk of bias and was rated as grade A. If only some of the criteria were met, the study was considered to have a moderate risk of bias and was rated as grade B. If a study did not meet any of the above criteria, it was considered to have a high risk of bias and was rated as grade C.

Study quality was also evaluated using the modified Jadad scale, which includes four domains: random sequence generation, allocation concealment, blinding, and withdrawals or dropouts. Based on the total score, studies scoring 1–3 points were classified as low quality, whereas those scoring 4–7 points were classified as high quality. Two researchers independently completed the quality assessment for each study. In case of disagreement, a third researcher was consulted to resolve differences until consensus was reached (Table 2).

### Statistical processing

Statistical analysis was performed using RevMan 5.4 software (The Cochrane Collaboration, Copenhagen, Denmark) and Stata 17.0 (64-bit) software. Continuous variables were analyzed using the standardized mean difference (SMD, Hedges’ g) with 95% confidence intervals 95% effect measures. Statistical heterogeneity was assessed using the I^2^ statistic. A fixed-effects model was used when statistical heterogeneity was low (*P* > 0.10 or I^2^ ≤ 50%). Otherwise, a random-effects model was applied. Subgroup analyses were performed to further explore sources of heterogeneity. When substantial heterogeneity was present among studies, sensitivity analyses were conducted using a leave-one-out approach to assess the robustness of the results.

### Sample size and power calculations

A priori power calculation for detecting a minimum clinically important difference of SMD = 0.30, with a two-sided *α* = 0.05 and 80% power, indicated that a total sample size of approximately 352 participants would be required, corresponding to the equivalent of about 9–10 moderately sized studies. *Post-hoc* considerations of statistical power were informed by the observed pooled effect size from the meta-analysis, which verify adequate power for detecting observed effects. For each included study, we recorded whether an a priori sample size or power calculation was reported. A sensitivity analysis was conducted to examine whether the study intensity affected the effect estimation.

### Measurement reliability assessment

For each outcome measure, we extracted reported test-retest reliability statistics (such as intraclass correlation coefficients (ICC), standard error of measurement (SEM), or minimal detectable change (MDC)) from the included studies, where available. Studies were rated on reliability reporting: (a) complete reliability data reported, (b) partial reliability data, (c) reliability not reported but cited, (d) no reliability information. This assessment was incorporated into the overall quality evaluation (See Appendix 3 for details).

With the exception of Yuan et al. (2022), all outcome measures were assessed using standardized methods that have been previously validated in the literature, as reported in the original studies (Table 2). (The detailed measurement specifications are explained in the Appendix 2 section.)

## Result

### Study selection reliability

Initial database searching identified 3,846 records. After duplicate removal, 3,263 unique records were screened. Independent screening was performed at the title/abstract and full-text stages. Disagreements between reviewers were rare and were resolved through discussion. For data extraction, independent extraction of a subset of studies showed high consistency, and all discrepancies were resolved by consensus.

### Literature search results

**Figure 3:**
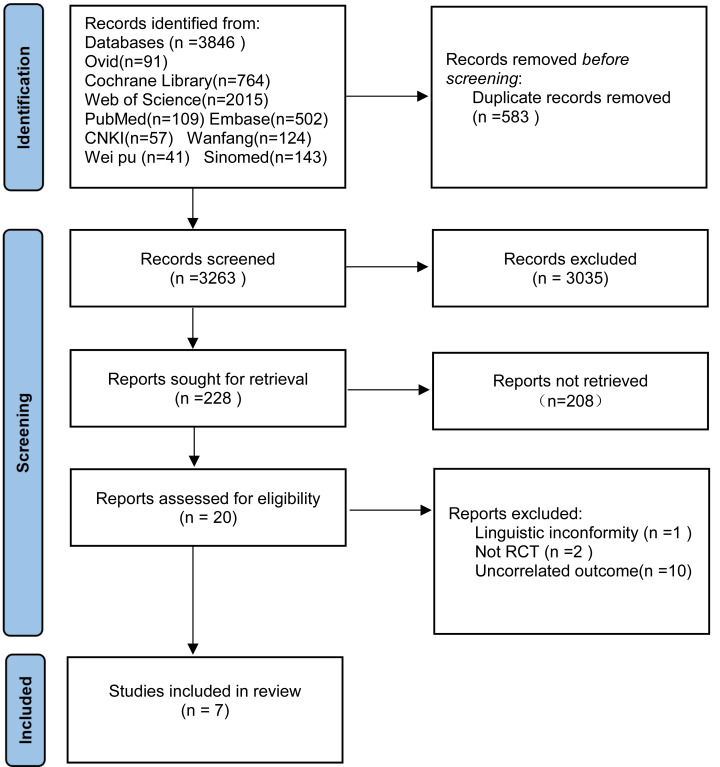
PRISMA flowchart.

Our initial search identified 3,846 potentially relevant articles across nine databases: Ovid (*n* = 91), Cochrane Library (*n* = 764), Web of Science (*n* = 2,015), PubMed (*n* = 109), Embase (*n* = 502), CNKI (*n* = 57), Wanfang (*n* = 124), VIP (*n* = 41), and Sinomed (*n* = 143). After importing all records into EndNote X9 and implementing our systematic screening protocol, seven studies met the final inclusion criteria (Fig. 3).

### Research characteristics and quality assessment

Summarizes the key features of the seven included randomized controlled trials, comprising a total of 246 CHF patients (intervention group: *n* = 122; control group: *n* = 124). The intervention duration was 12 weeks in six studies, while one study had an intervention period of 16 weeks (Tables 1 and 2).

### Measurement reliability

None of the included studies reported quantitative test–retest reliability for the primary outcome measures. In particular, no intraclass correlation coefficients (ICC) were provided for 6-minute walk distance (6WMD), maximal inspiratory pressure (PImax), or other functional outcomes. Although outcome measures were assessed using standardized methods that have been previously validated in the literature. However, the lack of formal reliability statistics indicates the methodological limitations of the current evidance base.

### Heterogeneity reporting enhancement

#### SPImax

Combined breathing training and aerobic exercise significantly improved SPImax compared to aerobic exercise alone (SMD = 2.43, 95% CI [−0.24 to 5.10], *p* = 0.07; *I*^2^ = 94.65%, *τ*^2^ = 3.52, p-heterogeneity = 0.00). The observed heterogeneity (*I*^2^ = 94.65%) indicates considerable between-study variance according to Cochrane guidelines warranting use of a random-effects model. However, since there were only two articles, subgroup analysis was not conducted (Appendix 4).

#### PImax

Combined breathing training and aerobic exercise significantly improved PImax compared to aerobic exercise alone (SMD = 0.29, 95% CI [−1.46 to 2.04], *p* = 0.75; *I*^2^ = 94.25%, *τ*^2^ = 2.24, p-heterogeneity = 0.00). The observed heterogeneity (*I*^2^ = 94.25%) indicates considerable between-study variance according to Cochrane guidelines warranting use of a random-effects model, and explore the sources of heterogeneity through subgroup analysis (Appendix 5).

#### Subgroup analyses

High heterogeneity was observed for the PImax outcome in the overall analysis. Therefore, subgroup analyses were conducted according to aerobic exercise training duration, age, and the proportion of male participants. The results showed significant differences between subgroups based on the the proportion of male participants. Compared with studies including a higher proportion of male participants, studies with a lower proportion of males demonstrated a significant effect (SMD = −1.45, 95% CI [−2.36 to −0.53], *p* < 0.001). Other factors did not show a clear moderating effect. However, given the limited number of included studies, these findings should be interpreted with caution (Table 3).

**Table 3:** Characteristics of the subgroup analysis.

**Subgroup**	**Studies (n)**	**I** ^**2**^ **(%)**	**Model**	**Effect size (95% CI)**	**P** **(within subgroup)**	**P** **(between subgroups)**
**PImax subgroup analysis**						
AT > 30 min	2	92.80%	Random-effects	−0.37 (−2.42, 1.69)	*P* < 0.001	*P* = 0.077
AT ≤ 30 min	1	0.00%	Random-effects	1.61 (0.87, 2.35)	*P* < 0.001	
Average age≥60	1	0.00%	Random-effects	1.61 (0.87, 2.35)	*P* < 0.001	*P* = 0.304
Average age<60	2	92.80%	Random-effects	−0.37 (−2.42, 1.69)	*P* < 0.001	
High proportion of males	2	73.50%	Random-effects	1.11 (0.17, 2.04)	*P* = 0.052	*P* = 0.502
Low proportion of males	1	0.00%	Random-effects	−1.45 (−2.36, −0.53)	*P* < 0.001	
**VE/VCO**_**2**_ ** slope subgroup analysis**						
12 weeks	3	93.60%	Random-effects	−0.53 (−1.82, 0.75)	*P* < 0.001	*P* = 0.077
16 weeks	1	0.00%	Random-effects	0.89 (−0.03, 1.80)	*P* < 0.001	
Average age≥60	2	95.70%	Random-effects	−0.76 (−2.67, 1.15)	*P* < 0.001	*P* = 0.304
Average age<60	2	65.30%	Random-effects	0.35 (−0.57, 1.26)	*P* = 0.09	
AT >30 min	3	94.20%	Random-effects	−0.34 (−1.84, 1.17)	*P* < 0.001	*P* = 0.502
AT ≤30 min	1	0.00%	Random-effects	0.22 (−0.42, 0.86)	*P* < 0.001	
High proportion of males	3	30.50%	Random-effects	0.25 (−0.23, 0.74)	*P* = 0.237	*P* = 0.000
Low proportion of males	1	0.00%	Random-effects	−1.72 (−2.19, −1.26)	*P* < 0.001	
**QoL subgroup analysis**						
12 weeks	4	83.90%	Random-effects	−1.15 (−1.97, −0.34)	*P* < 0.001	*P* = 0.576
16 weeks	1	0.00%	Random-effects	−0.81 (−1.71, 0.10)	*P* < 0.001	
AT >30 min	4	83.70%	Random-effects	−0.94 (−1.78, −0.10)	*P* < 0.001	*P* = 0.186
AT ≤ 30 min	1	0.00%	Random-effects	−1.70 (−2.46, −0.95)	*P* < 0.001	
Average age ≥60	2	0.00%	Random-effects	−1.80 (−2.20, −1.40)	*P* = 0.769	*P* = 0.005
Average age <60	3	63.10%	Random-effects	−0.60 (−1.34, 0.14)	*P* = 0.067	
High proportion of males	3	22.00%	Random-effects	−1.22 (−1.71, −0.72)	*P* = 0.278	*P* = 0.74
Low proportion of males	2	94.20%	Random-effects	−0.88 (−2.80, 1.04)	*P* < 0.001	

**Notes.**

marginal notes PImax maximal inspiratory pressure VE/VCO2 slope minute ventilationcarbon dioxide output slop QoL exercise time Quality of life AT Aerobic Exercise

Studies were stratified into high and low male proportion subgroups based on the median proportion of male participants across the included studies.

#### VE/VCO_2_ Slope

Combined breathing training and aerobic exercise significantly improved VE/VCO_2_ Slope compared to aerobic exercise alone (SMD = −0.20, 95% CI [−1.30 to 0.90], *p* = 0.72; *I*^2^ = 92.28%, *τ*^2^ = 1.15, p-heterogeneity = 0.00). The observed heterogeneity (*I*^2^ = 92.28%) indicates considerable between-study variance according to Cochrane guidelines warranting use of a random-effects model, and explore the sources of heterogeneity through subgroup analysis (Appendix 6).

#### Subgroup analyses

Subgroup analyses were performed for the VE/VCO_2_slope because substantial heterogeneity was observed. The analyses were conducted according to age, training duration in weeks, aerobic exercise duration, and the proportion of male participants. The subgroup analyses conducted for the high heterogeneity of the VE/VCO_2_ slope showed significant improvement only in the subgroup with a lower proportion of male participants (SMD = −1.72, 95% CI [−2.19 to −1.26], *p* < 0.001) (Table 3).

#### LVEF

Combined breathing training and aerobic exercise did not significantly improve LVEF compared to aerobic exercise alone (SMD = 0.12, 95% CI [−0.22 to 0.47], *p* = 0.49; *I*^2^ = 0.00%, p-heterogeneity = 0.58). The observed heterogeneity (*I*^2^ = 0.00%) indicates no significant heterogeneity; therefore, a fixed-effect model was used (Appendix 7).

#### LVESD

Combined breathing training and aerobic exercise did not significantly improve LVESD compared to aerobic exercise alone (SMD = −0.26, 95% CI [−0.65 to 0.12], *p* = 0.19; *I*^2^ = 39.30%, p-heterogeneity = 0.19). The observed heterogeneity (*I*^2^ = 39.30%) indicates low to moderate between-study variance according to Cochrane guidelines; therefore, a fixed-effect model was applied (Appendix 8).

#### LVEDD

Combined breathing training and aerobic exercise did not significantly improve LVEDD compared to aerobic exercise alone (SMD = 0.03, 95% CI [−0.36 to 0.41], *p* = 0.89; *I*^2^ = 0.00%, p-heterogeneity = 0.38). The observed heterogeneity (*I*^2^ = 0.00%) indicates no significant heterogeneity; therefore, a fixed-effect model was used (Appendix 9).

#### 6MWD

Combined breathing training and aerobic exercise did not significantly improve 6MWD compared to aerobic exercise alone (SMD = 0.28, 95% CI [−0.01 to 0.57], *p* = 0.06; *I*^2^ = 0.00%, p-heterogeneity = 0.59). The observed heterogeneity (*I*^2^ = 0.00%) indicates no significant heterogeneity; therefore, a fixed-effect model was used (Appendix 10).

#### Exercise time

**Figure 4:**
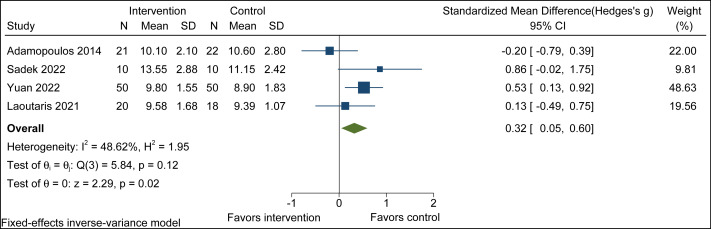
Exercise time forest plot. Note. Adamopoulos et al., 2014; Sadek et al., 2022; Yuan et al., 2022; Laoutaris et al., 2021.

Combined breathing training and aerobic exercise significantly improved Exercise time compared to aerobic exercise alone (SMD = 0.32, 95% CI [0.05 to 0.60], *p* = 0.02; *I*^2^ = 48.62%, p-heterogeneity = 0.12). The observed heterogeneity (*I*^2^ = 48.62%) indicates low to moderate between-study variance according to Cochrane guidelines; therefore, a fixed-effect model was applied (Fig. 4).

#### Quality of life

**Figure 5:**
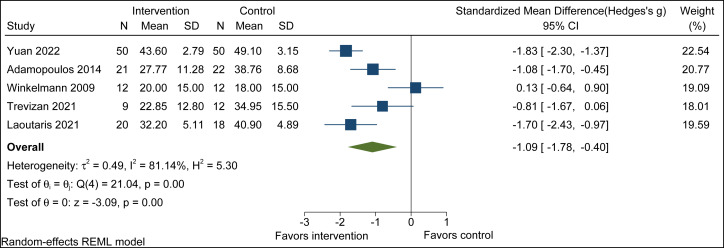
Quality of life forest plot. Note. Yuan et al., 2022; Adamopoulos et al., 2014; Winkelmann et al., 2009; Trevizan et al., 2021; Laoutaris et al., 2021.

Combined breathing training and aerobic exercise significantly improved QoL compared to aerobic exercise alone (SMD = −1.09, 95% CI [−1.78 to −0.40], *p* = 0.00; *I*^2^ = 81.14%, *τ*^2^ = 0.49, p-heterogeneity = 0.00). The observed heterogeneity (*I*^2^ = 81.14%) indicates considerable between-study variance according to Cochrane guidelines, a random-effects model was used, and explore the sources of heterogeneity through subgroup analysis (Fig. 5).

#### Subgroup analyses

Because substantial heterogeneity was observed in the effect size of this outcome, subgroup analyses were conducted based on the proportion of male participants, age, training duration in weeks, and Aerobic Exercise duration. The results suggested that age may be a moderating factor in quality of life improvement, with more pronounced and consistent benefits observed in patients aged ≥60 years following breathing training combined with aerobic exercise (SMD = −1.80, 95% CI [−2.20 to −1.40], *p* < 0.005) (Table 3).

### Sensitivity analysis

For outcomes with substantial heterogeneity, sensitivity analyses were performed using a leave-one-out approach.

For quality of life, the pooled effect remained statistically significant after exclusion of each individual study (*p* < 0.05), indicating good robustness of the results. Exclusion of the study by Winkelmann et al. (2009) markedly reduced heterogeneity, suggesting that this study was a major contributor to between-study variability, however, the direction of the overall effect remained unchanged.

For the VE/VCO_2_ slope, sensitivity analyses showed that the pooled effect did not reach statistical significance regardless of which study was excluded (*p* > 0.05), indicating a stable but non-significant overall effect. Notably, removal of the study by Yuan et al. (2022) substantially reduced heterogeneity, but did not alter the overall conclusion.

Sensitivity analysis for PImax indicated that the study by Winkelmann et al. (2009) substantially increased heterogeneity. After exclusion of this study, the pooled effect became statistically significant, suggesting a potential beneficial effect of the intervention that may be related to differences in patient characteristics. However, given the limited number of remaining studies and the presence of residual heterogeneity, this finding should be interpreted with caution.

## Discussion

CHF is a complex clinical syndrome that is commonly considered the end stage of various cardiovascular diseases. It is characterized by structural and functional abnormalities of the heart, leading to reduced cardiac output and substantial impairment of patients’ functional status and quality of life. To reduce morbidity and mortality in patients with CHF (Mcvannel et al., 2024; Skouri et al., 2025), exercise training and breathing training have gradually become important adjunctive components of cardiac rehabilitation (Laoutaris et al., 2021).

Previous studies have shown that aerobic exercise can improve cardiac and vascular function by suppressing sympathetic activity and increasing peripheral blood flow (Hambrecht et al., 2000; Chircop & Jelinek, 2006). In addition, Breathing training can enhance inspiratory muscle function and strength in patients with CHF and improve hemodynamics at rest and during exercise (Larsen et al., 2002; Corrà et al., 2002; Dall’ago et al., 2006). Moreover, some studies have concluded that combining Breathing training with Aerobic Exercise can further improve cardiopulmonary exercise capacity and enhance quality of life in patients with CHF (Adamopoulos et al., 2014).

However, the available evidence is limited by small sample sizes and inconsistent results. This study systematically evaluated the effects of Breathing training combined with Aerobic Exercise in patients with CHF through a systematic review and meta-analysis, providing more comprehensive evidence to support the clinical application of this combined intervention strategy.

### Clinical significance and minor clinical differences

Beyond statistical significance, the pooled effects were further interpreted from a clinical perspective. For the six-minute walk distance (6WMD), although the pooled effect size did not reach statistical significance (SMD = 0.28, 95% CI [−0.01 to 0.57]), the mean differences reported in the original studies showed that the intervention group all improved by more than 35 m in most studies. This magnitude exceeds the established minimal clinically important difference (MCID) for patients with chronic heart failure (25–30 m). Therefore, these findings suggest that the combined intervention may have potential clinical relevance for functional endurance, although the stability of this effect should be interpreted with caution.

For left ventricular ejection fraction (LVEF), the pooled results showed a small magnitude of improvement (SMD = 0.12, *p* = 0.49). In most included studies, the absolute changes did not reach the commonly accepted threshold of 5% for clinical relevance, suggesting that improvements in this parameter may be limited at the clinical level.

With respect to quality of life (QoL), the pooled analysis demonstrated a significant improvement, and the magnitude of change in some studies approached or exceeded the established minimal clinically important difference (MCID) of the respective scales. This indicates that the combined intervention may have clear clinical value in patient-reported outcomes.

Overall, the combined intervention demonstrated potential clinical relevance for functional endurance and quality of life, whereas its effects on cardiac structural and functional parameters require further confirmation in high-quality studies. This patient-centered interpretation of outcomes is consistent with previous recommendations for the assessment of clinical relevance (Böhme Kristensen, Asimakopoulou & Scambler, 2023).

### Synergistic effects of breathing training combined with aerobic exercise on pulmonary function in patients with CHF

In patients with chronic heart failure, pulmonary function is an important indicator for assessing disease severity, treatment outcomes, exercise capacity, and quality of life (Mangner et al., 2013). Through a systematic review, the present study found that breathing training combined with aerobic exercise showed a tendency to improve pulmonary function in patients with chronic heart failure.

From a mechanistic perspective, improvements in inspiratory muscle function may play a key role by attenuating the inspiratory muscle metaboreflex. In patients with chronic heart failure, inspiratory muscle fatigue can activate the metaboreflex, leading to sympathetic activation, peripheral vasoconstriction, and reduced limb perfusion, which further exacerbates exertional dyspnea (Hossein Pour et al., 2020). In contrast, inspiratory muscle training can reduce activation of the respiratory muscle metaboreflex by increasin inspiratory muscle strength and fatigue resistance (Lima et al., 2025). This can improve blood supply to peripheral muscles and attenuate excessive increases in mean arterial pressure and heart rate, thereby reducing symptoms of dyspnea (Chiappa et al., 2008).

In addition, studies by Ponikowski et al. (1997) have shown that patients with chronic heart failure commonly exhibit increased peripheral chemoreceptor sensitivity, autonomic imbalance, and impaired baroreflex function. Enhanced ventilatory drive can lead to a disproportionate ventilatory response during exercise. This mechanism provides an important neurophysiological explanation for excessive ventilation and exertional dyspnea in patients with heart failure, even in the absence of overt hypoxemia. Meanwhile, previous studies have shown that inspiratory muscle training can reduce excessive ventilatory demand during exercise by attenuating peripheral chemoreflex activity and sympathetic activation, thereby improving ventilatory efficiency. This may provide an important neurophysiological basis for the effects of breathing training combined with aerobic exercise on improving pulmonary function and cardiopulmonary exercise capacity in patients with chronic heart failure (Tumminello et al., 2007).

Through subgroup analysis, it was shown that in the subgroup with a higher proportion of males, the combined training resulted in a greater improvement in PImax. This finding may be related to sex-related differences in expiratory muscle mass, thoracic mechanical characteristics, or strength adaptability. In addition, the observed effect may partly be attributable to specific respiratory muscle weakness present at baseline in the study population. However, these findings were derived from exploratory subgroup analyses with a limited number of included studies and should therefore be interpreted with caution. Further large-scale, high-quality randomized controlled trials are needed to confirm the synergistic effects of breathing training and aerobic exercise on lung function in patients with CHF.

### Synergistic effects of breathing training combined with aerobic exercise on cardiac function in patients with CHF

Left ventricular ejection fraction (LVEF) is an important indicator of myocardial contractile function (Olson et al., 2010), whereas left ventricular end-systolic diameter (LVESD) and left ventricular end-diastolic diameter (LVEDD) are commonly used to assess left ventricular structure and the degree of ventricular remodeling (Piepoli et al., 2004; Ribeiro, Chiappa & Callegaro, 2012). The results of the present study showed that breathing training combined with aerobic exercise did not produce significant improvements in LVEF, LVESD, or LVEDD. This finding is consistent with the results reported by Li et al. (2025). Previous studies have shown that exercise training can significantly improve exercise tolerance and physical aspects of quality of life in patients with chronic heart failure. However, it does not produce significant changes in LVEF or other cardiac function parameters, suggesting that the main benefits of exercise interventions may arise from peripheral adaptations rather than structural cardiac remodeling (Piepoli et al., 2004).

Edwards et al. (2023) reported in a meta-analysis that, compared with traditional moderate-intensity training, high-intensity interval training was more effective in improving cardiopulmonary exercise capacity in patients with heart failure. However, the effects of different exercise modalities on left ventricular structure and function were generally limited. Possible reasons for these findings include the high sensitivity of left ventricular dimensions and ejection fraction to inter-individual variability and measurement error, making small short-term changes difficult to detect (Piepoli et al., 2004). In addition, the beneficial effects of exercise training on left ventricular remodeling may depend on the stimulus intensity and the duration of the intervention. In the included studies, the intervention period ranged from 12 to 16 weeks, which may have been too short to induce significant structural cardiac changes between groups (Ribeiro, Chiappa & Callegaro, 2012).

Moreover, aerobic exercise and breathing training may have overlapping physiological effects on improving peripheral circulation and neural regulation. As a result, their combination may not produce additive effects in the short term (Ribeiro, Chiappa & Callegaro, 2012). In addition, measurements of LVEF and ventricular dimensions rely on echocardiography, and differences in measurement techniques and equipment may also contribute to variability in the results.

### Synergistic effects of breathing training combined with aerobic exercise oncardiopulmonary exercise capacity in patients with CHF

The results of the present study indicate that breathing training combined with aerobic exercise can improve cardiopulmonary exercise capacity in patients with chronic heart failure. However, the effects were not consistent across different cardiopulmonary exercise capacity indicators. The combined intervention showed a significant improvement in exercise time, whereas the overall effects on the 6MWD and the VE/VCO_2_ slope did not reach statistical significance.

The underlying mechanism may be related to the “blood steal” phenomenon affecting the inspiratory muscles during exercise. Impaired inspiratory muscle function can accelerate fatigue in the limb skeletal muscles, thereby reducing exercise endurance in patients (Roveda et al., 2003). Breathing training and aerobic exercise may act synergistically to promote improvements in cardiopulmonary exercise capacity. As noted by Lima in a review, adjunctive breathing training can reduce the blood flow demand of the respiratory muscles while increasing blood flow distribution to skeletal muscles, thereby enhancing muscle fatigue resistance by reducing reliance on anaerobic metabolism (Lima et al., 2025). At the same time, aerobic exercise can induce adaptive changes in skeletal muscle structure, metabolism, and neural regulation, thereby improving overall exercise efficiency and reducing metabolic stress (Oliva et al., 2023). The complementary effects of these two training modalities at the peripheral level may jointly reduce ventilatory burden during exercise, enhance lung compliance, and promote overall improvements in cardiopulmonary function in patients with chronic heart failure (Ruiling & Weiping, 2024; Sadek et al., 2024).

Subgroup analyses conducted to address the high heterogeneity of the VE/VCO_2_ slope showed that a significant improvement was observed only in subgroups with a lower proportion of male participants, suggesting that sex composition may be an important factor influencing the intervention effects. Sex may influence the ventilatory efficiency response to the intervention, with female patients potentially exhibiting greater plasticity in ventilatory control. However, these results should be interpreted with caution, and further validation is needed in studies with larger sample sizes and more balanced population characteristics.

### Synergistic effects of breathing training combined with aerobic exercise on quality of life in patients with CHF

Quality of life is an important outcome for evaluating symptom relief and overall well-being in patients with chronic heart failure. Because of the high prevalence, recurrent symptoms, and high mortality of the disease, quality of life is often severely impaired in these patients (Baodi et al., 2018). All five studies included in the present analysis used the Minnesota Living with Heart Failure Questionnaire (MLHFQ) to evaluate the effects of breathing training combined with aerobic exercise on quality of life in patients with chronic heart failure. The results showed that breathing training combined with aerobic exercise significantly improved quality of life in patients with chronic heart failure, which is consistent with the findings of the meta-analysis by Gomes Neto et al. (2018).

Previous studies have shown that combined improvements at both physiological and psychological levels may jointly contribute to enhanced quality of life in patients with chronic heart failure. On the one hand, improvements in quality of life are associated with enhanced cardiovascular and respiratory function in patients. Regular training can reduce blood pressure and heart rate, thereby improving stroke volume and cardiac output. These effects help alleviate symptoms of heart failure. In addition, regular training may improve sleep disturbances, enhance appetite, and reduce the negative impact of the disease, leading to a significant improvement in overall quality of life in patients with chronic heart failure (Shu, 2023; Zhou et al., 2023; Savarese et al., 2023). On the other hand, breathing training combined with aerobic exercise may help regulate hypothalamic–pituitary–adrenal axis function and reduce levels of stress-related hormones, such as adrenocorticotropic hormone. This may alleviate negative psychological states, promote psychological adaptation, and thereby improve quality of life in patients (Smart & Steele, 2011).

Age may also play a moderating role in improvements in quality of life, possibly because patients with CHF aged ≥60 years tend to have lower baseline quality of life and more severe symptoms, leaving greater room for improvement following the combined intervention. In addition, the MLHFQ is more sensitive to interventions targeting symptom burden, fatigue, and dyspnea, and older patients may experience more pronounced improvements in these domains.

### Comparison with other studies and implications for practice

The present study adopted the systematic comparison framework proposed by Albertoni et al. (2023) to examine how different interventions act across multiple physiological systems in chronic heart failure. Within this framework, optimized pharmacological therapy does not directly increase exercise capacity, but instead alleviates exercise intolerance and symptoms by suppressing excessive neuroendocrine activation and improving cardiopulmonary hemodynamics, endothelial function, and peripheral perfusion, thereby reducing ventilatory overactivation and muscular abnormalities (Mcvannel et al., 2024; Skouri et al., 2025). Although guideline-directed medical therapy (GDMT) improves clinical outcomes, its real-world implementation remains limited, as interindividual variability, comorbidities, and drug tolerance often prevent patients from achieving target doses.

Device-based therapies, such as cardiac resynchronization therapy (CRT), can substantially modify cardiac hemodynamics and autonomic balance, leading to improved cardiac function and outcomes in selected patients; however, their effects on symptoms and functional status vary across individuals (Herr et al., 2014). Exercise-based interventions exert distinct systemic effects. High-intensity interval and resistance training primarily enhance aerobic capacity and peripheral muscle strength (Segizbaeva et al., 2015). In contrast, breathing training combined with aerobic exercise mainly improves respiratory muscle function, modulates neural reflexes, and reduces ventilatory demand. Owing to its relatively low training load, this approach may be particularly suitable for patients with chronic heart failure and limited exercise tolerance, serving as a complementary option to existing therapeutic strategies.

From an implementation perspective, breathing training is a feasible and low-cost adjunctive intervention. A meta-analysis demonstrated that inspiratory muscle training significantly improves peak oxygen uptake, maximal inspiratory pressure, ventilatory efficiency, 6MWD, dyspnea, and quality of life in stable patients with chronic heart failure, supporting its clinical efficacy and safety (Yang et al., 2025). Compared with device-based therapies or high-intensity supervised exercise programs, breathing training requires minimal equipment and can be delivered in outpatient or home settings and easily integrated into standard cardiac rehabilitation programs (Piepoli et al., 2004). Training for breathing training mainly focuses on the correct use of breathing techniques, appropriate training intensity, and monitoring during the intervention (Piepoli et al., 2010a).

Appropriate patient selection remains essential. Patients with marked exercise intolerance, poor ventilatory efficiency, or respiratory muscle weakness—particularly those unable to participate in high-intensity training—may derive greater benefit from breathing training (Piepoli et al., 2010b). When applied in clinically stable patients with appropriate supervision and monitoring, breathing training represents a safe and practical adjunct to pharmacological therapy and other rehabilitation approaches (Piepoli et al., 2010a; Yang et al., 2025).

### Limitations

First, the number of included studies was limited (*k* = 7), and the total sample size was relatively small. This may have resulted in insufficient statistical power, limited the feasibility of subgroup analyses and increased the risk of publication bias.

Second, substantial heterogeneity was observed among the included studies in breathing training protocols, including training modality (inspiratory threshold loading *vs.* resistive breathing devices), training intensity, and intervention duration. In addition, aerobic exercise prescriptions varied considerably in intensity, exercise modality (cycling, walking, or combined training), and level of supervision. This methodological inconsistency limited the ability to clearly analyze dose–response relationships and may have contributed to increased between-study heterogeneity.

Third, the intervention duration in most of the included studies was relatively short (ranging from 12 to 16 weeks), which may have limited the magnitude of the effects observed for certain outcomes.

Fourth, only published studies were included in the present analysis, and unpublished or grey literature was not retrieved, which may have increased the risk of publication bias. In addition, restricting inclusion to English-language publications may have introduced language bias.

Fifth, most included studies provided unclear reporting on allocation concealment and blinding of outcome assessment, which may have led to an overestimation of effect sizes, particularly for subjective outcomes such as quality of life. In addition, no study provided test–retest reliability data for the primary outcome measures, nor did any study report patient satisfaction with the intervention.

Sixth, there were notable differences in patient characteristics among the included studies, including the severity of chronic heart failure (as reflected by LVEF), sex distribution, pharmacological treatment regimens, and comorbid conditions, which may limit thegeneralizability of the findings.

Future randomized controlled trials with larger sample sizes, longer follow-up durations,more standardized intervention protocols, and more rigorous methodological reporting are needed to further confirm and extend the findings of the present study.

## Conclusion

This systematic review and meta-analysis indicate that breathing training and aerobic exercise may have synergistic beneficial effects on multiple functional outcomes in patients with CHF.

The combined intervention showed an overall trend toward improvement in inspiratory muscle function, and its effects may be influenced by baseline patient characteristics, training intensity, and sex composition. In contrast, the combined intervention showed no significant effects on left ventricular ejection fraction or ventricular structural parameters, suggesting that its benefits are mainly mediated through improvements in peripheral function and neural regulatory adaptations rather than short-term cardiac structural remodeling. In addition, breathing training and aerobic exercise improved cardiopulmonary exercise capacity, and the magnitude of improvement may be influenced by sex composition. With respect to quality of life, the intervention demonstrated stable and significant improvements, which were more pronounced in older patients.

Overall, breathing training and aerobic exercise appear to be a safe and feasible non-pharmacological intervention with potential synergistic effects. Future large-scale randomized controlled trials with long-term follow-up are needed to further determine the optimal training parameters and appropriate target populations.

##  Supplemental Information

10.7717/peerj.20954/supp-1 Supplemental Information 1 Search strategy

10.7717/peerj.20954/supp-2 Supplemental Information 2 Measurement method

10.7717/peerj.20954/supp-3 Supplemental Information 3 PRISMA checklist

10.7717/peerj.20954/supp-4 Supplemental Information 4 SPImax forest plot

10.7717/peerj.20954/supp-5 Supplemental Information 5 PI max forest plot

10.7717/peerj.20954/supp-6 Supplemental Information 6 VE/VCO Slope forest plot

10.7717/peerj.20954/supp-7 Supplemental Information 7 LVEF forest plot

10.7717/peerj.20954/supp-8 Supplemental Information 8 LVEDD forest plot

10.7717/peerj.20954/supp-9 Supplemental Information 9 LVESD forest plot

10.7717/peerj.20954/supp-10 Supplemental Information 10 6WMD forest plot

10.7717/peerj.20954/supp-11 Supplemental Information 11 Data organization in the literature

## Additional Information and Declarations

### Data Availability

The following information was supplied regarding data availability: This is a meta-analysis.
