# Supplementary material for: Synergistic effects of combined breathing training and aerobic exercise on cardiopulmonary function in chronic heart failure: a systematic review and meta-analysis
Source: PeerJ. 2026 Mar 18;14:e20954. doi: 10.7717/peerj.20954 (PMC13005616; doi:10.7717/peerj.20954)
Supplement: Supplemental Information 1 [file peerj-14-20954-s001.docx]

Database：PubMed

Search conducted: [Database establishment will be completed by April 2025]

#1."Breathing Exercises"[Mesh]

#2.("Breathing Exercises"[Title/Abstract] OR "Respiratory Muscle Training"[Title/Abstract] OR "breathing therapy"[Title/Abstract] OR "chest physical therapy"[Title/Abstract] OR "chest physiotherapy"[Title/Abstract] OR "respiration exercise"[Title/Abstract] OR "respiration therapy"[Title/Abstract] OR "respiratory exercise"[Title/Abstract] OR "respiratory physiotherapy"[Title/Abstract] OR "breathing exercise"[Title/Abstract] OR "respiratory muscle training"[Title/Abstract])

#3."Exercise"[Mesh]

#4.("Exercise"[Title/Abstract] OR "Exercises"[Title/Abstract] OR "Physical Exercise*"[Title/Abstract]OR "Aerobic Exercise*"[Title/Abstract] OR "Isometric Exercise*"[Title/Abstract] OR "Acute Exercise*"[Title/Abstract] OR "Exercise Training*"[Title/Abstract] OR "Physical Activity"[Title/Abstract] OR "Physical Activities"[Title/Abstract])

#5."Heart Failure"[Mesh]

#6("Heart Failure"[Title/Abstract] OR "Cardiac Failure"[Title/Abstract] OR "Congestive Heart Failure"[Title/Abstract] OR "Heart Decompensation"[Title/Abstract] OR "Myocardial Failure"[Title/Abstract] OR "cardiac backward failure"[Title/Abstract] OR "cardiac decompensation"[Title/Abstract] OR "Cardiac Failure"[Title/Abstract] OR "cardiac incompetence"[Title/Abstract] OR "cardiac insufficiency"[Title/Abstract] OR "(cardiac stand still"[Title/Abstract] OR "cardial decompensation[Title/Abstract]" OR "cardial insufficiency"[Title/Abstract] OR "chronic heart failure"[Title/Abstract] OR "chronic heart insufficiency"[Title/Abstract] OR "Congestive Heart Failure"[Title/Abstract] OR "decompensatio cordis"[Title/Abstract] OR "heart backward failure"[Title/Abstract] OR "heart decompensation"[Title/Abstract] OR "heart incompetence"[Title/Abstract] OR "heart insufficiency[Title/Abstract]" OR "insufficientia cardis"[Title/Abstract] OR "left sidedleft sidedmyocardial failure"[Title/Abstract] OR "myocardial failure"[Title/Abstract] OR "myocardial insufficiency"[Title/Abstract])

#7.#1 OR #2

#8.#3 OR #4

#9.#5 OR #6

10.#7 AND #8 AND #9

Database：Embase

Search conducted: [Database establishment will be completed by April 2025]

1.'breathing exercise'/exp

2.'breathing exercises':ab,ti OR 'breathing therapy':ab,ti OR 'chest physical therapy':ab,ti OR 'chest physiotherapy':ab,ti OR 'respiratory physiotherapy':ab,ti OR 'breathing exercise':ab,ti OR 'respiration exercise':ab,ti OR 'respiration therapy':ab,ti OR 'respiratory exercise':ab,ti

3.'exercise'/exp

4.exercise:ab,ti OR 'biometric exercise':ab,ti OR 'effort':ab,ti OR 'exercise capacity':ab,ti OR 'exercise performance':ab,ti OR 'exercise training':ab,ti OR 'exertion':ab,ti OR 'fitness training':ab,ti OR 'fitness workout':ab,ti OR 'exercise':ab,ti OR 'physical effort':ab,ti OR 'physical exercise':ab,ti OR 'physical exertion':ab,ti OR 'physical workout':ab,ti

5.'heart failure'/exp

6.'cardiac backward failure':ab,ti OR 'cardiac decompensation':ab,ti OR 'cardiac failure':ab,ti OR 'cardiac incompetence':ab,ti OR 'cardiac insufficiency':ab,ti OR 'cardiac stand still':ab,ti OR 'cardial decompensation':ab,ti OR 'cardial insufficiency':ab,ti OR 'chronic heart failure':ab,ti OR 'chronic heart insufficiency':ab,ti OR 'decompensatio cordis':ab,ti OR 'heart backward failure':ab,ti OR 'heart decompensation':ab,ti OR 'heart incompetence':ab,ti OR 'heart insufficiency':ab,ti OR 'insufficientia cardis':ab,ti OR 'myocardial failure':ab,ti OR 'myocardial insufficiency':ab,ti OR 'heart failure':ab,ti

7.#1 OR #2

8.#3 OR #4

9.#5 OR #6

10.#7 AND #8 AND #9

数据库：Cochrane

Search conducted: [Database establishment will be completed by April 2025]

1.MeSH descriptor: [Breathing Exercises] explode all trees

2.'Breathing Exercises' OR 'Respiratory Muscle Training' OR ' Training, Respiratory Muscle' OR ' Muscle Training, Respiratory' OR ' Exercise, Breathing'

3.MeSH descriptor: [Exercise] explode all trees

4‘Exercise' OR ' Isometric Exercise*' OR ' Physical Activities' OR ' Exercise Training*' OR ' Physical Activity' OR ' Exercises' OR ' Physical Exercise' OR ' Aerobic Exercise*' OR ' Acute Exercise*' OR ' Physical Exercises'.
5.MeSH descriptor: [Heart Failure] explode all trees

6.‘Heart Failure' OR ' Myocardial Failure' OR ' Congestive Heart Failure' OR ' Cardiac Failure' OR ' Right-Sided Heart Failure' OR ' Left-Sided Heart Failure' OR ' Left Sided Heart Failure' OR ' Right Sided Heart Failure'

7.#1 OR #2

8.#3 OR #4

9.#5 OR #6

10.#7 AND #8 AND #9

数据库：Web of Science

Search conducted: [Database establishment will be completed by April 2025]

1."Breathing Exercises (Topic) OR Exercise, Breathing (Topic) OR Respiratory Muscle Training (Topic) OR Muscle Training, Respiratory (Topic) OR Training, Respiratory Muscle (Topic) "

2."Exercise (Topic) OR Exercises (Topic) OR Exercise Training (Topic) OR Exercise Trainings (Topic)OR Physical Exercise (Topic) OR Physical Exercises (Topic) OR Physical Activity (Topic) OR AerobicExercise (Topic) OR Aerobic Exercises (Topic) OR Isometric Exercises (Topic) OR Isometric Exercise (Topic) OR Acute Exercise* (Topic) OR Physical Activities (Topic) "

3."Heart Failure (Topic) OR Cardiac Failure (Topic) OR Heart Decompensation (Topic) OR Myocardial Failure (Topic) OR Congestive Heart Failure (Topic) OR Left-Sided Heart Failure (Topic) OR Left Sided Heart Failure (Topic) OR Right Sided Heart Failure (Topic) "

4."#3 AND #2 AND #1 "
