# Supplementary material for: Synergistic effects of combined breathing training and aerobic exercise on cardiopulmonary function in chronic heart failure: a systematic review and meta-analysis
Source: PeerJ. 2026 Mar 18;14:e20954. doi: 10.7717/peerj.20954 (PMC13005616; doi:10.7717/peerj.20954)
Supplement: Supplemental Information 2 [file peerj-14-20954-s002.docx]

**Measuring tools**

**SPImax**

**Adamopoulos et al., 2014,**Sustained maximal inspiratory pressure was measured using an electronic manometer and dedicated computer software (Model: **TRAINAIR®**; pressure accuracy: ±0.1%; Manufacturer: **Project Electronics Ltd**; Country: **London, UK**). The methodology for PImax measurement was validated based on the protocols described by **Ionescu AA et al.** (Am J Respir Crit Care Med, 1998;158:1271–1276) and **Laoutaris I et al.** (Eur J Cardiovasc Prev Rehabil, 2004;11:489–496).

**Laoutaris et al., 2021,**PImax was measured using an electronic manometer and computer software with a pressure accuracy of ±0.1% (Model: **TRAINAIR®**; Manufacturer: **Project Electronics Ltd**; Country: **London, UK**). The methodology for PImax measurement was validated according to the protocol described by **Laoutaris ID et al.** (Int J Cardiol, 2013;167:1967–1972).

**VE/VCO_2_ slope**

**Adamopoulos et al., 2014,**The VE/VCO₂ slope was measured using gas exchange analysis equipment, including the Medgraphics CPX/MAX (manufactured by Medical Graphics Corp., St. Paul, MN, USA) in Athens, the Schiller CS200® (manufactured by Schiller AG, Baar, Switzerland) in Hasselt, and the Oxycon Pro® (manufactured by Jaeger-Toennies, Höchberg, Germany) in Bern. On the morning of each test day, gas and volume calibrations were executed. The measurement method for the VE/VCO₂ slope was verified by Bosnac-Guclu M et al. (Resp Med 2011; 105: 1671–1681).

**Trevizan et al., 2021,**the VE/VCO₂ slope was measured using a pulmonary function/gas exchange analysis system (Vmax Encore 29 System; manufactured by VIASYS Healthcare Inc., Yorba Linda, CA, USA) with a computerized breath-by-breath analysis system. The exercise load was provided by a cycle ergometer (Via Sprint 150P; manufactured by Ergoline, Bitz, Germany). The measurement method for the VE/VCO₂ slope was verified by Antunes-Correa LM et al. (J Cachexia Sarcopenia Muscle 2020; 1: 89–102), Skinner JS et al. (Res Q Exerc Sport 1980; 1: 234–248), and Chua TP et al.(J Am Coll Cardiol 1997; 7: 1585–1590).

**Laoutaris et al., 2021,**The VE/VCO₂ slope was measured using a combination of gas exchange analysis and exercise load equipment, including the Medgraphics CPX/MAX (manufactured by Medical Graphics Corp., St. Paul, MN, USA) and the ZAN 600 computerized breath-by-breath spiroergometry system (manufactured by ZAN Messgerate GmbH, Germany) coupled with a Schiller treadmill (Carrollton, USA).Pulmonary gas exchange was analysed breath by breath and averaged every 10 seconds. Gas and volume calibrations were performed on the morning of each test day. The measurement method for the VE/VCO₂ slope was verified by Riley M et al. (Eur Heart J 1992; 13: 1363–1367) and Balady GJ et al. (Champaign, IL: Human Kinetics; 2004. p79).

**Wang et al., 2022,**The VE/VCO₂ slope was determined using the Schiller CS200 cardiopulmonary exercise cycle ergometer to assess the subjects’ exercise capacity and pulmonary function. (The model, sampling frequency, manufacturer, and country of origin of the relevant equipment were not mentioned)Static lung volume measurements were performed prior to each cardiopulmonary exercise test.

**PI_max_**

**Adamopoulos et al., 2014**,PImax was measured using an electronic manometer and dedicated computer software (Model: TRAINAIR®; Pressure Accuracy: ±0.1%; Manufacturer: Project Electronics Ltd; Country: London, UK). The methodology for PImax measurement was validated with reference to the protocols established by Ionescu AA et al. (Am J Crit Care Med 1998; 158:1271–1276) and Laoutaris I et al. (Eur J Cardiovasc Prev Rehabil 2004; 11:489–496).

**Winkelmann et al., 2009**,PImaxas measured using a pressure transducer (Model: MVD-500 V.1.1 Microhard System; Manufacturer: Globalmed; Country: Porto Alegre, Brazil). The measurement methodology was validated with reference to the protocol reported by Dall'Ago P et al. (J Am Coll Cardiol 2006; 47:757-63) for inspiratory muscle training in patients with heart failure and inspiratory muscle weakness.

**Laoutaris et al., 2021,**PImax was measured using an electronic manometer and computer software with a pressure accuracy of ±0.1% (Model: TRAINAIR®; Manufacturer: Project Electronics Ltd; Country: London, UK). The methodology for PImax measurement was validated based on the protocol described by Laoutaris ID et al. (Int J Cardiol 2013; 167:1967–1972).

**Exercise time**

**Adamopoulos et al., 2014,**Exercise time was assessed using an electronically braked cycle ergometer in Hasselt and Bern: the device used in Hasselt was manufactured by Ergofit GmbH & Co (Pirmasens, Germany), whereas the device used in Bern was the Ergometrics 800S model, produced by Ergoline® GmbH (Bitz, Germany). A cycling cadence of 70 revolutions per minute (r.p.m.) was maintained for all tests. The methodology for exercise time assessment was validated based on the protocol described by Riley M et al. (Eur Heart J 1992; 13:1363–1367).

**Sadek et al., 2022**,Exercise time was evaluated using the Bruce treadmill protocol, with no specific equipment parameters reported.

**Laoutaris et al., 2021**,Exercise time was measured using a combination of gas exchange analysis and exercise load equipment, including the Medgraphics CPX/MAX (manufactured by Medical Graphics Corp., St. Paul, MN, USA) and the ZAN 600 computerized breath-by-breath spiroergometry system (manufactured by ZAN Messgerate GmbH, Germany) coupled with a Schiller treadmill (Carrollton, USA). The measurement method for the Exercise time was verified by Riley M et al. (Eur Heart J 1992; 13: 1363–1367) and Balady GJ et al. (Champaign, IL: Human Kinetics; 2004. p79).

**Wang et al., 2022,**The Exercise time was determined using the Schiller CS200 cardiopulmonary exercise cycle ergometer to assess the subjects’ exercise capacity and pulmonary function. (The model, sampling frequency, manufacturer, and country of origin of the relevant equipment were not mentioned)Static lung volume measurements were performed prior to each cardiopulmonary exercise test.

LVEF,LVESD,LVEDD

**Trevizan et al., 2021,**LVEF was measured using two-dimensional echocardiography with the Simpson method (Device model: IE33; Manufacturer: Philips Medical Systems; Location: Andover, MA, USA), and the measurement method was validated by Lang RM et al. (Lang RM 2015; 3: 233–270).

**Adamopoulos et al., 2014,**LVEF、LVESD、LVEDD were measured by standard two-dimensional resting echocardiography using the following devices: Ultrasound Vivid 7 (manufacturer: General Electric Healthcare, Fairfield, CT, USA) and Sequoia C512 (manufacturer: Siemens Medical Solutions, Mountain View, CA, USA).The biplane Simpson’smethod in an apical four-chamber view was used to estimate LVEF (%),while LV end-diastolic (LVEDD, mm) and end-systolic diameter (LVESD,mm) were measured using the Teichholz method.

**Laoutaris et al., 2021,**LVEF、LVESD、LVEDD were assessed using standard two-dimensional resting echocardiography with the devices of Ultrasound Vivid 7 or 6 (manufacturer: General Electric Healthcare, Fairfield, CT, USA).The biplane Simpson’s method in an apical four-chamber view was used to estimate LVEF (%), while LVEDD (mm) and LVESD (mm) were measured using the Teichholz method.

**Sadek et al., 2024,**LVEF、LVESD、LVEDD were measured via the echocardiographic method using a Vivid S6 ultrasound device (manufacturer: General Electric Healthcare, USA).Biplane Simpson’s method (apical four-chamber view) was used for the estimation of echocardiographic measures. Parameters assessed during echocardiography were left ventricular ejection fraction (LVEF in %), left ventricular end systolic/ diastolic diameters (LVESD and LVEDD in mm).

**6MWD**

**Laoutaris et al., 2021,**Six-minute walk distance was assessed following American Thoracic Society guidelines (ATS, 2002) in a 30-meter corridor（There is no specific information about the instrument）

**Wang et al., 2022,**Select a corridor with a length of 30m in the ward to set up a starting point. Place a chair at each starting point. Under the monitoring of medical staff, the patient walks at the maximum speed he can tolerate for 6 minutes, and measures the walking distance, i.e. 6min walking distance (6MWD).

**Winkelmann et al., 2009,**Six-minute walk distance was assessed following the guidelines by Olsson LG et al. (Olsson LG, 2005) for the evaluation of treatment outcomes in randomized, blinded intervention trials of chronic heart failure (There is no specific information about the instrument).

**Sadek et al., 2024,**Six-minute walk test was assessed following the guidelines by Beatty AL et al. (Beatty AL, 2012) as a prognostic tool in stable coronary heart disease.The test was performed in a 60-m corridor under the supervision of a physical therapist.

**Quality of life**

**Wang et al., 2022**，Quality of life was assessed with the Minnesota Living withHeart Failure Questionnaire.

**Adamopoulos et al., 2014，** Quality of life was assessed using the MLwHFQ,which was validated by Rector TS, et al. (Rector TS 1987; 3: 198–209).

**Winkelmann et al., 2009，Laoutaris et al., 2021,Trevizan et al., 2021,** Quality of life was assessed with the Minnesota Living withHeart Failure Questionnaire.which was validated by Rector TS et al.(Rector TS 1992; 4: 1017–1025)
