# Supplementary figures and images for: Synergistic effects of combined breathing training and aerobic exercise on cardiopulmonary function in chronic heart failure: a systematic review and meta-analysis
Source: PeerJ. 2026 Mar 18;14:e20954. doi: 10.7717/peerj.20954 (PMC13005616; doi:10.7717/peerj.20954)

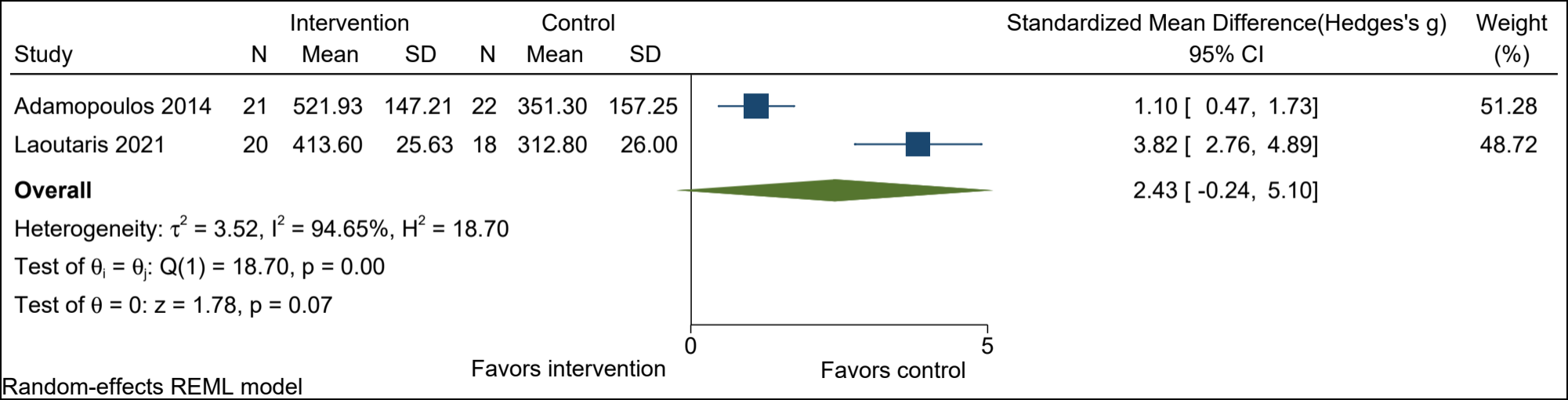

Supplement: Supplemental Information 4 [file peerj-14-20954-s004.pdf]

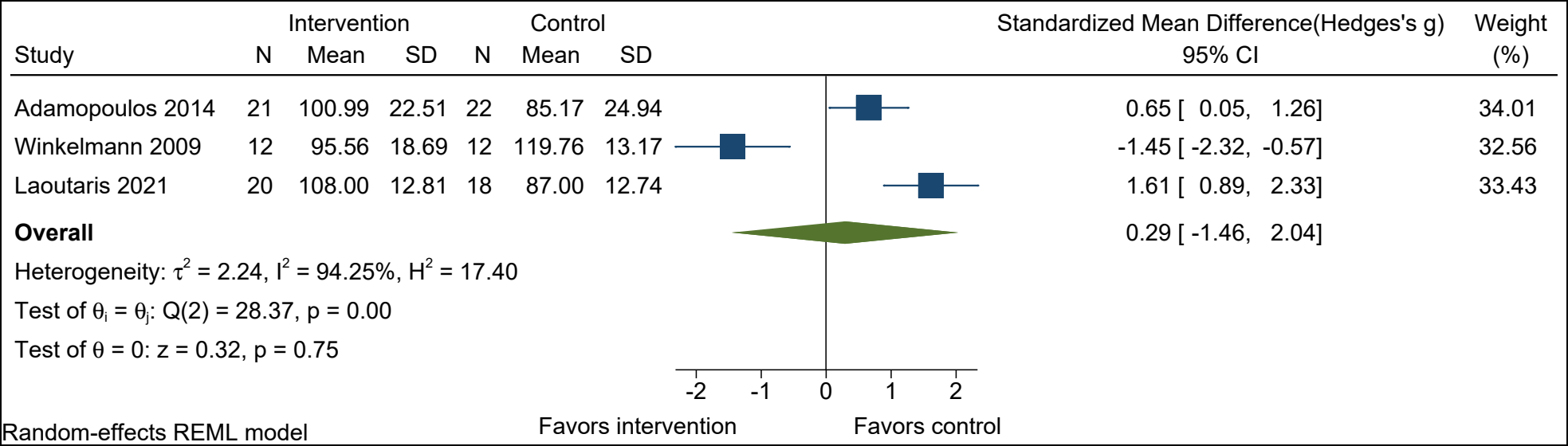

Supplement: Supplemental Information 5 [file peerj-14-20954-s005.pdf]

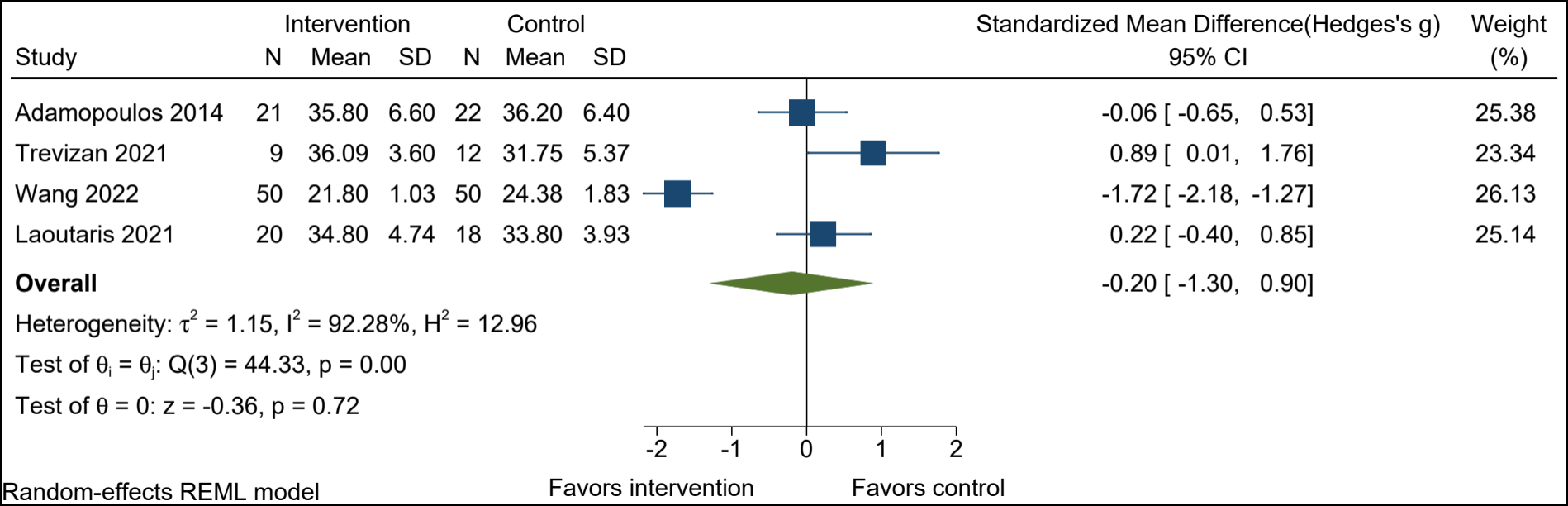

Supplement: Supplemental Information 6 [file peerj-14-20954-s006.pdf]

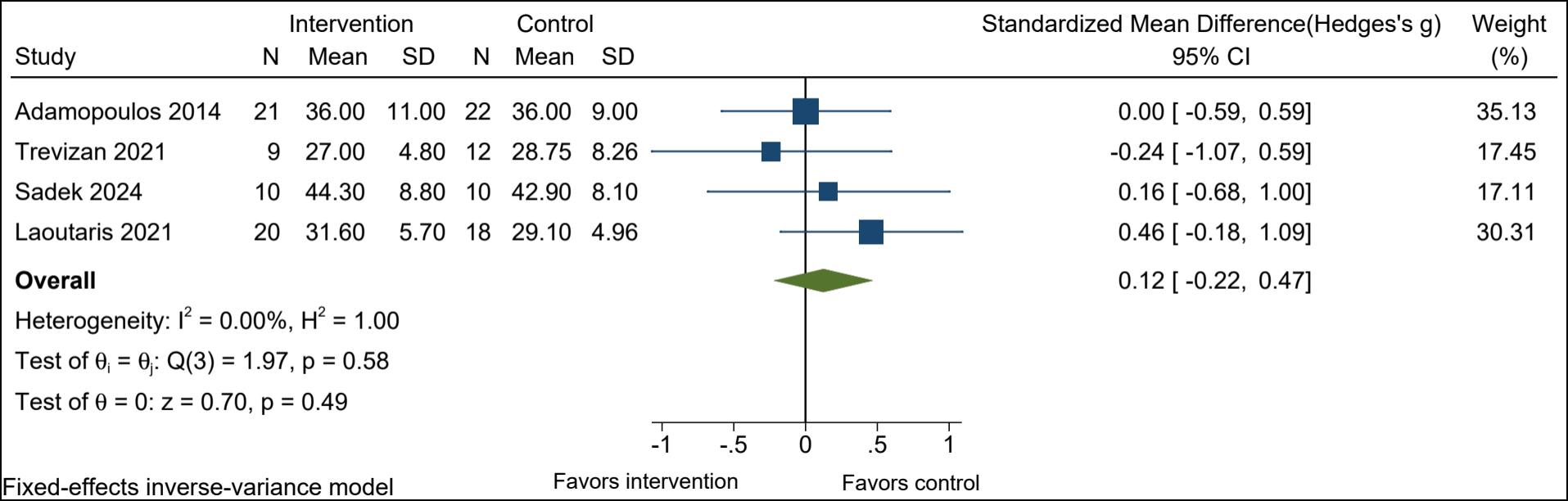

Supplement: Supplemental Information 7 [file peerj-14-20954-s007.pdf]

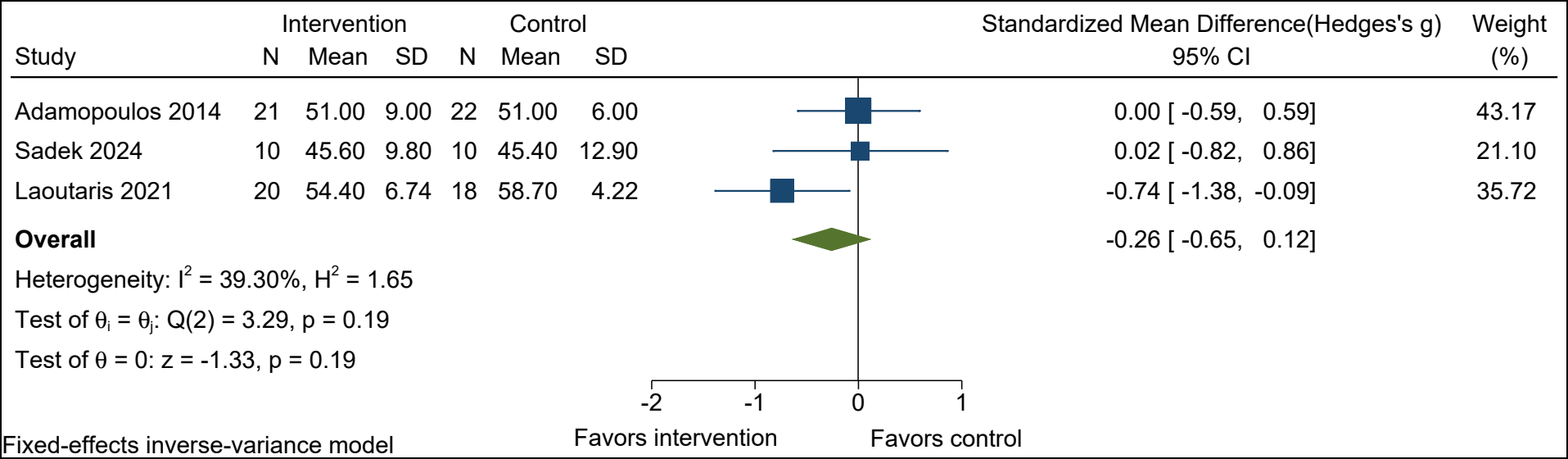

Supplement: Supplemental Information 8 [file peerj-14-20954-s008.pdf]

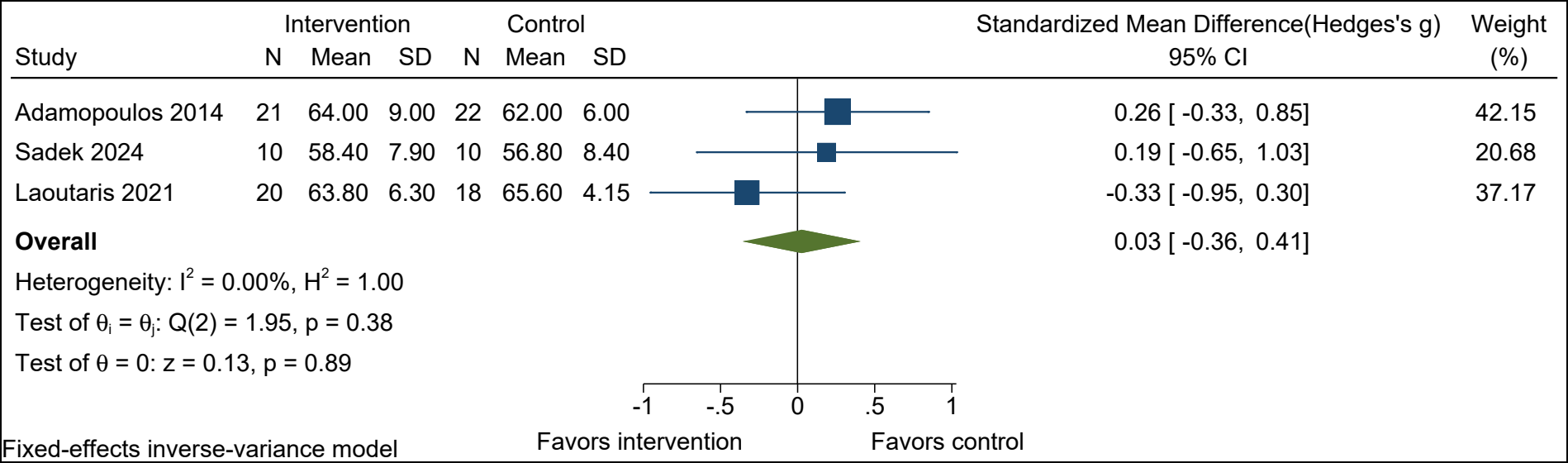

Supplement: Supplemental Information 9 [file peerj-14-20954-s009.pdf]
