# Supplementary material for: Synergistic effects of combined breathing training and aerobic exercise on cardiopulmonary function in chronic heart failure: a systematic review and meta-analysis
Source: PeerJ. 2026 Mar 18;14:e20954. doi: 10.7717/peerj.20954 (PMC13005616; doi:10.7717/peerj.20954)
Supplement: Supplemental Information 10 [file peerj-14-20954-s010.pdf]

Study	Intervention N	Intervention Mean	Intervention SD	Control N	Control Mean	Control SD	Standardized Mean Difference(Hedges's g) 95% CI	Weight (%)
Sadek 2024	10	567.50	75.90	10	491.50	170.00	0.55 [-0.30, 1.41]	11.27
Wang 2022	50	374.60	36.56	50	362.54	23.17	0.39 [-0.00, 0.78]	53.63
Winkelmann 2009	12	500.00	72.00	12	489.00	81.00	0.14 [-0.63, 0.91]	13.82
Laoutaris 2021	20	452.20	53.33	18	455.50	62.81	-0.06 [-0.68, 0.57]	21.28
**Overall**							0.28 [-0.01, 0.57]	

Heterogeneity: $I^2 = 0.00\%$, $H^2 = 1.00$

Test of $\theta_i = \theta_j$: Q(3) = 1.94, p = 0.59

Test of $\theta = 0$: z = 1.90, p = 0.06

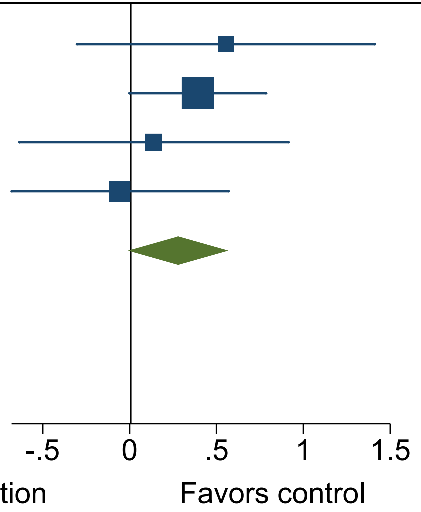

Fixed-effects inverse-variance model
